# The Effects of Fibrinogen’s Interactions with Its Neuronal Receptors, Intercellular Adhesion Molecule-1 and Cellular Prion Protein

**DOI:** 10.3390/biom11091381

**Published:** 2021-09-18

**Authors:** Nurul Sulimai, Jason Brown, David Lominadze

**Affiliations:** 1 Department of Surgery, Morsani College of Medicine, University of South Florida, Tampa, FL 33612, USA; nurulsulimai@usf.edu (N.S.); jasonb3@usf.edu (J.B.); 2 Department of Molecular Pharmacology and Physiology, Morsani College of Medicine, University of South Florida, Tampa, FL 33612, USA

**Keywords:** mitochondrial activation, neuronal death, proinflammatory cytokine, nitrite, proximity ligation, ROS

## Abstract

Neuroinflammatory diseases, such as Alzheimer’s disease (AD) and traumatic brain injury (TBI), are associated with the extravascular deposition of the fibrinogen (Fg) derivative fibrin and are accompanied with memory impairment. We found that during the hyperfibrinogenemia that typically occurs during AD and TBI, extravasated Fg was associated with amyloid beta and astrocytic cellular prion protein (PrP^C^). These effects coincided with short-term memory (STM) reduction and neurodegeneration. However, the mechanisms of a direct Fg–neuron interaction and its functional role in neurodegeneration are still unclear. Cultured mouse brain neurons were treated with Fg in the presence or absence of function-blockers of its receptors, PrP^C^ or intercellular adhesion molecule-1 (ICAM-1). Associations of Fg with neuronal PrP^C^ and ICAM-1 were characterized. The expression of proinflammatory marker interleukin 6 (IL-6) and the generation of reactive oxygen species (ROS), mitochondrial superoxide, and nitrite in neurons were assessed. Fg-induced neuronal death was also evaluated. A strong association of Fg with neuronal PrP^C^ and ICAM-1, accompanied with overexpression of IL-6 and enhanced generation of ROS, mitochondrial superoxide, and nitrite as well as the resulting neuronal death, was found. These effects were reduced by blocking the function of neuronal PrP^C^ and ICAM-1, suggesting that the direct interaction of Fg with its neuronal receptors can induce overexpression of IL-6 and increase the generation of ROS, nitrite, and mitochondrial superoxide, ultimately leading to neuronal death. These effects can be a mechanism of neurodegeneration and the resultant memory reduction seen during TBI and AD.

## 1. Introduction

Fibrinogen (Fg) is a one of the most abundant plasma proteins with its main physiological function playing a role in hemostasis. The hemostasis is a result of Fg’s conversion to a fibrin monomer and its subsequent aggregation that can mediate clot formation [1]. In cases of head trauma commonly accompanied with hemorrhage, Fg is thought to have a critical role in hemostasis. Interestingly, the Fg derivative, fibrin, has been found in postmortem brain sections taken from patients anywhere from hours up to as long as 18 years after a fall injury [2]. Furthermore, the deposition of Fg in extravascular spaces [3], particularly between a blood vessel and astrocyte endfeet [4], coincided with increased neurodegeneration and a reduction in short-term memory (STM) during mild-to-moderate traumatic brain injury (m-mTBI) [4,5]. Fg is a high (~340 kD) molecular weight glycoprotein with a tri-nodular structure of about 46 nm in length [1,6] and a Stokes–Einstein radius of about 8.4 nm [7] (e.g., Stokes–Einstein radius of bovine serum albumin is ~3.5 nm [6,8]). Thus, during normal physiological conditions with a blood content of ~2 mg/mL [9], Fg remains in the circulation. However, during an inflammatory disease such as m-mTBI, which is associated with an increased blood level of Fg and with a compromised blood–brain barrier, even without vascular ruptures, Fg gains access to the extravascular space [4,10,11,12]. Due to its large Stokes–Einstein radius, slight openings of endothelial junctions are not sufficient to allow Fg (as opposed to albumin) to cross the vascular wall. The main path of its inflammation-associated extravasation is via caveolar transcytosis [4,10,11,12].

Cellular prion protein (PrP^C^) is a glycosylphosphatidylinositol-anchored glycoprotein abundantly expressed on the surfaces of neurons [13], while intercellular adhesion molecule-1 (ICAM-1) is a transmembrane glycoprotein that is constitutively expressed at low levels in unstimulated endothelial cells [14] and neurons [15,16]. Both PrP^C^ [17] and ICAM-1 [18,19] are known Fg receptors. An increase in ICAM-1 expression was attributed to stimulation by inflammatory cytokines and oxidant species [20,21]. Similarly, increased expression of PrP^C^ was seen during experimental m-mTBI [17]. An association of Fg with extravascular proteins, e.g., with PrP^C^, could lead to the formation of degradation resistant protein complexes [22] that can cause neurodegeneration [3,4,10]. PrP^C^ has been argued to play a dual role in the nervous system: providing neuroprotection and mediating toxic signaling [23]. Among many of its effects, PrP^C^ has been shown to function as a neurotoxic protein [23]. Thus, possible activation of PrP^C^ on the surface of neurons may itself cause neurotoxicity and thus result in neuronal death seen in the present study.

Recently, we have shown that Fg induced the activation of astrocytes, upregulating astrocytic inflammatory cytokines and generating reactive oxygen species (ROS) that affected the viability of neurons in an astrocyte-neuron co-culture system [24]. These effects were a result of Fg’s association with its astrocytic receptors PrP^C^ and ICAM-1 [24]. Higher neuronal apoptosis was found in neurons co-cultured with Fg-activated astrocytes [24]. In injured brains, it is likely that extravasated Fg can come into direct contact with neurons, causing some functional changes in these cells. Later, when Fg is converted to fibrin, resulting in its accumulation in the brain, further destructive effects can be manifested. We have found that neurodegeneration and the resultant reduction in STM was associated with the m-mTBI, which was accompanied with an elevated level of Fg [3,4]. However, the precise mechanisms of Fg-induced neuronal death are not clear.

The aim of the present study was to investigate if Fg can directly interact with its receptors PrP^C^ and ICAM-1 on the surface of neurons, similar to what we have observed with the astrocytes [24]. We have shown that both PrP^C^ [17] and ICAM-1 [25] positively act as Fg receptors on astrocytes [24]. Based on these results and data by others [15,16,18,21], we hypothesized that Fg would associate with its neuronal receptors PrP^C^ and ICAM-1, resulting in the activation of inflammatory responses such as the overexpression of interleukin-6 (IL-6), the generation of ROS, and neuronal death. The study revealed that an interaction between Fg and neurons induced an increase in the expression of the proinflammatory cytokine IL-6, enhanced oxidative damage, and neuronal death, in part due to its direct association with neuronal PrP^C^ and ICAM-1.

## 2. Materials and Methods

### 2.1. Experimental Setups and Groups

Neurons were used either on day 7, 10 or 13 of growth. Since neurons express thrombin [26], each experimental group contained hirudin (0.5 U/mL) to prevent any possible effect of thrombin-induced conversion of Fg into fibrin, unless indicated. Neurons were grown in primary neuron basal Medium™ (PNBM; Lonza, Basel, Switzerland) containing primary neuron growth medium (PNGM) SingleQuots^TM^ (Lonza, Basel, Switzerland) growth supplements that include neural survival factor-1 and glial cell outgrowth suppressant (cat. # CC-4461; Lonza), which are serum-free and do not contain thrombin. The same media were used for cell treatment. On the day of the experiment, half of the old media were mixed with fresh PNGM neuron media. Neurons were treated with 0.5 mg/mL of Fg, 1 mg/mL of Fg, 1 mg/mL of Fg in the presence of 10 μg/mL of the prion protein-blocking peptide, or 10 μg/mL of the function-blocking antibody against ICAM-1. Control cells were treated with a medium containing phosphate-buffered saline (PBS) in volume equal to that of Fg. As a positive control, 200 ng/mL of TNFα was used with co-stimulation with 200 ng/mL of a murine IFNγ. The cells were kept in an incubator at 37 °C with 5% CO_2_/air and treated for 2 or 24 has indicated.

### 2.2. Cell Culture

Primary mouse brain cortex neurons from C57BL/6 mice (cat. #M-cx-300) purchased from Lonza, were plated at 7 × 10^5^ cells/mL. The neurons were grown in PNBM and PNGM^TM^ Single Quots^TM^ as recommended by the manufacturer. Neurons were grown in 8-well removeable chambers from IBIDI (Gräfelfing, Germany) or 24-well plates from Nunc™ (Thermo Fisher Scientific, Waltham, MA, USA) with a #1 glass coverslip for cell immunocytochemistry and image analysis, 24-well plates from Nunc™ (Rochester, NY, USA) for gene analysis, and 12-well plates (Nunc™) for neuronal IL-6 protein level detection. The cell culture plates were coated with Poly-d-Lysine (30 µg/mL) and laminin (200 µg/mL) for neuronal growth.

### 2.3. Materials and Reagents

The polyclonal rabbit antibody against human Fg (cross-reacts with mouse) was purchased from Dako Cytomation (Carpentaria CA, USA). The rat purified function-blocking antibody (clone: YN1/1.7.4) against mouse ICAM-1 (CD-54, cat. # 116133) was obtained from BioLegend (San Diego, CA, USA), and the prion protein-blocking peptide (GTX89339-PEP) was from GeneTex (Irvine, CA, USA). For detection of ICAM-1 and PrP^C^, we used antibodies against ICAM-1/CD54 raised in mouse (cat. # NBP2-22541, Novus Biologicals, Littleton, CO, USA) and against prion protein, also raised in mouse (cat. # P0110, Sigma Aldrich Chemicals Co., St. Louis, MO, USA), respectively. Poly-d-Lysine, laminin, hirudin, lipopolysaccharides from *Escherichia coli* (O111:B4), and Duolink^®^ In Situ Detection Reagent Red were from Sigma. Recombinant murine tumor necrosis factor alpha (TNFα) (cat. # 315-01A-20UG) and interferon gamma (IFNγ) (cat. # 315-05-20UG) were purchased from Peprotech (Rocky Hill, NJ, USA). PBS (composition: 1.05 mM of KH_2_PO_4_, 155.17 mM of NaCl, and 2.97 mM of Na_2_HPO_4_. 7H2O, without Ca^2+^ and Mg^2+^), Hanks’ balanced salt solution (HBSS) with Ca^2+^ and Mg^2+^, and TRIzol reagent were from Invitrogen (Carlsbad, CA, USA). Carboxy-2′,7′-dichlorodihydrofluorescein diacetate (H2DCFDA) Image-IT™ LIVE Green-ROS detection kit and MitoSOX™ Red-mitochondrial superoxide indicator were purchased from Thermo Fisher Scientific.

### 2.4. Proximity Ligation Assay (PLA)

To evaluate whether Fg associates with and can form complexes with PrP^C^ and ICAM-1 on the surfaces of neurons, we used PLA as we performed a similar study on Fg and its astrocytic receptors [24]. This method allowed us to visualize individual ligand–receptor interactions at a single-molecule resolution. Neurons first were rinsed with PBS and then fixed with 4% paraformaldehyde in PBS for 15 min and permeabilized with 0.05% TritonX-100 for 10 min. PLA was performed following the protocol suggested by the manufacturer and as described in [24]. Briefly, the cells were incubated with Duolink^®^ blocking solution overnight at 4 °C. We investigated the association of Fg with PrP^C^ and Fg with ICAM-1 in two parallel sets of experiments. PLA probes with anti-Fg (1:400) and anti-PrP^C^ (1:100) or anti-ICAM-1 (1:150) were used. Immunoglobulin G (IgG) was used as an isotype control for antibodies against PrP^C^ and ICAM-1. Neurons were treated with Fg but probed with IgG as a replacement for one of the primary antibodies in the PLA to validate the assay. The neurons were first incubated with the antibody pairs for 1.5 h at 37 °C (primary antibody incubation) and after washing they were incubated with Duolink^®^ PLA probes (secondary antibody incubation) for 1 h at 37 °C. The primary antibodies act as antigens to the PLA oligonucleotide-conjugated plus and minus probes. The plus and minus probes serve as secondary antibodies against the two host species of the primary antibodies (anti-mouse and anti-rabbit) and are conjugated with plus and minus Duolink ^®^ PLA probes (1:5). Cells were washed twice with wash buffer prior to amplification steps. Probed neurons were incubated with the Duolink^®^ ligation-ligase solution (30 min at 37 °C) followed by incubation with the amplification-polymerase solution (100 min at 37 °C). Then, cells were washed with another wash buffer and mounted with the Duolink^®^ PLA mounting medium with 4′,6-diamidino-2-phenylindole (DAPI). PLA signals were present only when Fg was within the proximity of less than 40 nm of its pair, PrP^C^ or ICAM-1. The signals were identified as fluorescent spots (red, λexcitation/λemission 598/634 nm).

### 2.5. Quantitative Real-Time PCR (qRT-PCR)

RNA was isolated from the neuron cultures using TRIzol reagent according to the manufacturer’s (Invitrogen) instruction. Total RNA from each sample was used for cDNA synthesis using an iScript cDNA synthesis kit from Bio-Rad (Hercules, CA, USA), following the manufacturer’s instruction. qRT-PCR analysis was carried out using PowerUp™ SYBR™ Green Master Mix (Applied Biosystems, Austin, TX, USA). The PCR cycle parameters were: 50 °C for 2 min, followed by 95 °C for 10 min, then 40 cycles at 95 °C for 15 s, and the annealing temperatures were kept between 56 and 60 °C for 1 min, according to optimized annealing temperature for the different set of primers. Gene expression levels were determined by QuantStudio 3 from Life Technologies (Carlsbad, CA, USA). The mRNA expression of target gene was analyzed and normalized to 18S, which was used as the housekeeping gene. Data analysis of fold changes in gene expression was perfomed using the ΔΔCt method and presented as 2^−(average ΔΔCt)^. The following primers were used: IL-6-Fwd 5′-GACTTCCATCGAGTTGCCTTCT-3′, Rev 5′-TTGGGAGTGGTATCCTCTGTGA-3′, and 18S-Fwd 5′-CGGCGACGACCCATTCGAAC-3′, Rev 5′-GAATCGAACCCTGATTCCCCGTC-3′.

### 2.6. Enzyme-Linked Immunosorbent Assay (ELISA)

The level of IL-6 in the neuronal conditioned medium was measured by sandwich ELISA using a commercial Mouse IL-6 ELISA kit (cat. # ab46100) from Abcam (Cambridge, MA, USA) according to the manufacturer’s recommendation.

### 2.7. ROS, MitoSOX and Nitrite Detection

Generation of neuronal nitrate, mitochondrial superoxide, and intracellular ROS were detected using a Griess assay (Promega, Madison, WI, USA), MitoSOX™ red mitochondrial superoxide indicator (Invitrogen, Waltham, MA, USA), and carboxy-H2DCFDA Image-IT™ LIVE Green ROS Detection Kit (Invitrogen), respectively, following manufacturers’ recommendations. The use of carboxy-H_2_DCFDA, which is cell permeable and becomes fluorescent when the acetate groups are removed by intracellular esterases and oxidation occurs within the cells, enables us to investigate intracellular ROS, whereas mitSOX selectively detects superoxide production in the mitochondria. MitoSOX accumulates in mitochondria due to its positive charge and reliably detects relative differences in mitochondrial ROS production in cells [27]. For the ROS assay, cells were incubated with carboxy-H2DCFDA at a final concentration of 30 μM in HBSS with Ca^2+^ and Mg^2+^ at 37 °C for 30 min in the dark. To detect mitochondrial superoxide, cells were stained with mitoSOX at a final concentration of 5 μM in HBSS with Ca^2+^ and Mg^2+^ and incubated at 37 °C for 10 min. MitoSOX-dyed cells were counterstained with DAPI to show the cells’ nuclei. Fluorescence intensity was measured at the wavelength of 494/529 nm (λexcitation/λemission) for ROS and 510/580 nm for mitoSOX. The mean fluorescence intensity ratio was presented after subtraction of the background fluorescence. Nitrite for Griess assay was measured using an absorbance filter between 520 and 550 nm with Biotek Synergy H1 plate reader (BioTek Instruments Inc., Winooski, VT, USA).

### 2.8. LIVE/DEAD^®^ Viability/Cytotoxicity Assay

LIVE/DEAD^®^ Viability/Cytotoxicity Assay (Thermo Fisher Scientific) was performed as recommended by the manufacturer. It discriminates live cells from the dead by simultaneously activating green-fluorescing calcein-AM (494/517 nm) that indicates intracellular esterase activity and red-fluorescing ethidium homodimer-1 (EthD-1) (528/617 nm) that indicates dead cells with a loss of plasma membrane integrity. Calcein-AM and EthD-1 were used at working concentrations of 2 and 2.5 µM in HBSS (with Ca^2+^ and Mg^2+^), respectively.

### 2.9. Image Analysis

To detect proximity ligation of target proteins, cells were observed using a laser-scanning confocal microscope Olympus FV1000 (Shinjuku City, Tokyo, Japan) with a 60X objective. Z-stacks of a selected constant-size area of interests (AOI) in each experimental well were acquired. The microscope settings were kept constant for all images to allow an adequate comparison. Deconvolution of images were carried out using Olympus CellSens Dimension Desktop 2.3 (Olympus Corporation, Shinjuku City, Tokyo, Japan). Acquired images were analyzed with ImageJ (version 1.52a) and the number of PLA-positive signals were counted using the Image-based Tool for Counting Nuclei plugin function in the ImageJ software. PLA signals per cell in 7 randomly selected AOIs were analyzed and averaged for each treatment group.

For the mitoSOX and ROS assays, images were taken using an Olympus FV1000 confocal microscope. For live/dead cell assay, images were taken using Olympus IX51 fluorescence microscope (Tokyo, Japan). During the entire image acquisition duration, the microscope settings were kept constant for each experiment in order to allow an adequate comparison. Data were normalized per number of cells in image.

### 2.10. Statistical Analysis

The obtained data were analyzed using Graph Pad Prism software (San Diego, CA, USA). All data were expressed as the mean ± SEM. The experimental groups were compared by a one-way analysis of variance (ANOVA). If the ANOVA indicated a significant difference (*p* < 0.05), a Bonferroni’s post hoc test was used to compare group means.

## 3. Results

### 3.1. Fg Increased Death of Neurons

We found that Fg increased neuronal death both in the presence and absence of the hirudin that was used to prevent the conversion of Fg to fibrin (Figure S1). Neuronal cell death was not influenced by the presence of hirudin, suggesting that soluble Fg and fibrin had similar effects on neurons. All the other experiments were performed in the presence of hirudin.

**Figure 1:**
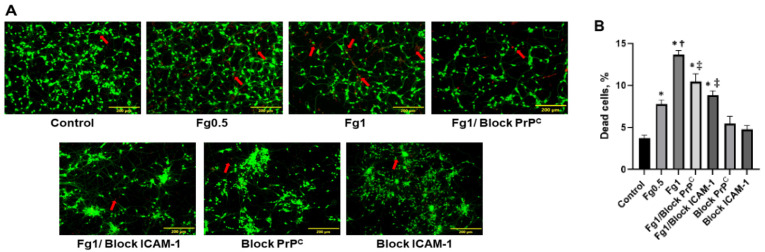
Neuronal cell death induced by fibrinogen (Fg). (**A**) Representative images show staining of live/dead neurons that were treated with medium alone (control), 0.5 mg/mL of Fg (Fg0.5), 1 mg/mL of Fg (Fg1), 1 mg/mL of Fg (Fg1) in the presence of a cellular prion protein (PrP^C^)-blocking peptide (Fg1/block PrP^C^), 1 mg/mL of Fg in the presence of a function-blocking antibody against intercellular adhesion molecule-1 (ICAM-1) (Fg1/block ICAM-1), the prion protein-blocking peptide alone, or the ICAM function-blocking antibody alone. Hirudin was present in all experimental groups to prevent the conversion of Fg to fibrin. (**B**) The summary of the image analyses for the detection of live neurons using a live/dead assay. An automatic cell count was performed based on the fluorescence signal threshold of the images provided by the OLYMPUS CellSens Dimension Desktop 2.3. Arrows indicate exemplary dead cells shown in red. Data are presented as the average of number of dead cells as a percent of the total number of cells in a selected constant area of interest in each experimental group. *p* < 0.05; ∗—vs. Control, †—vs. Fg0.5, ‡—vs. Fg1; n = 4.

Fg dose-dependently increased neuronal cell death (Figure 1). Blocking the function of either PrP^C^ or ICAM-1 reduced Fg-induced cell death (Figure 1).

### 3.2. The Association of Fg with PrP^C^ and ICAM-1 on the Surface of Neurons

**Figure 2:**
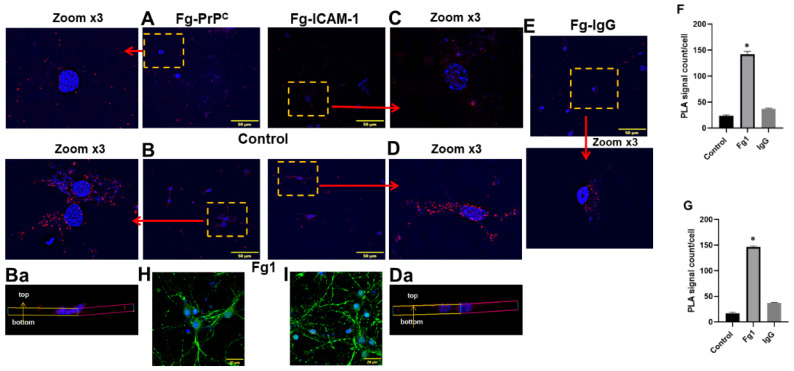
Interactions of fibrinogen (Fg) with its receptors, cellular prion protein (PrP^C^) and intercellular adhesion molecule-1 (ICAM-1) on the surface of neurons detected by proximity ligation assay (PLA). Representative images of PLA signals shown as red dots indicating interactions between (**A**,**B**) Fg and PrP^C^ (Fg-PrP^C^) and (**C**,**D**) between Fg and ICAM-1 (Fg-ICAM-1). Neurons were treated with medium alone (control) (**A**,**C**) or with 1 mg/mL of Fg (Fg1) for 24 h (**B**,**D**). Fg, PrP^C^ and ICAM-1 were detected using anti-Fg, anti-PrP^C^, and anti-ICAM-1 antibodies, respectively. Enlarged (3× zoom) images (shown in yellow punctuated boxes) are pointed to by red arrows. Standardized size images of each selected area of interest were collected by acquiring at least 4 focal planes with increments of 0.5 µm to form a final z-stock image. Representative three-dimensional reconstructions of the PLA signals (Fg-PrP^C^ and Fg-ICAM-1) on the surface of neurons from the respective z-stack images (Ba,Da). (**E**) One group of neurons treated with 1 mg/mL of Fg were probed with Immunoglobulin G (IgG) (Fg-IgG) and used as a negative control to define the specificity of the PrP^C^ and ICAM-1 detecting antibodies. Cellular nuclei were labeled with 4′,6-diamidino-2-phenylindole shown in blue in all images. (**F**,**G**) Summary of data analysis of interaction of Fg with PrP^C^ and ICAM-1, respectively. The number of PLA signals per cell was quantified into 7 randomly selected similar size areas of interest in each image and averaged for each treatment group. (**H,I**) Neurons were treated similar to **B**,**D** using PLA to detect an interaction of Fg with PrP^C^ (**H**) and with ICAM-1 (**I**) with the addition of receiving counterstaining with microtubule-associated protein 2 (green) as a neuronal marker. Cellular nuclei were labeled with 4′,6-diamidino-2-phenylindole (blue). Hirudin was present in all experimental groups to prevent the conversion of Fg to fibrin. *p* < 0.05 in all; ∗—vs. Control; *n* = 4.

The specific associations between Fg and its neuronal receptors PrP^C^ and ICAM-1 were detected with PLA in two separate sets of experiments: one to define an interaction of Fg with PrP^C^ (Figure 2A,B,Ba,F) and another to define an interaction of Fg with ICAM-1 (Figure 2C,D,Da,G) on the surfaces of neurons. The quantification of the PLA signal density per cell corresponded to the molecular interactions observed between Fg and its receptors under study. Data showed that there were significantly higher interactions between Fg and both of its receptors on neurons compared to those in the control group (untreated cells). The interaction between Fg and the IgG, isotype control (non-specific neuronal receptor) was significantly lower than its interactions with its receptors (Figure 2E). Furthermore, representative 3D reconstructions of the PLA signals from z-stack images demonstrated that the PLA signals were formed all along the neurons (Figure 2Ba,Da). In addition, we incorporated technical controls within the PLA by individually omitting each of the primary antibodies to detect any possible non-specific binding (Figure S2). The lack of PLA signals in any of the three negative controls (Figure S2) suggested a high specificity of the used antibodies and validated the PLA in the present study. As an additional control test, neurons in PLA were counterstained with the microtubule-associated protein 2 (MAP2) to specifically identify them (Figure 2H,I). Images clearly show that the PLA signal was from MAP-2-positive cells, i.e., neurons.

### 3.3. Fg-Induced Upregulation of IL-6 in Neurons

**Figure 3:**
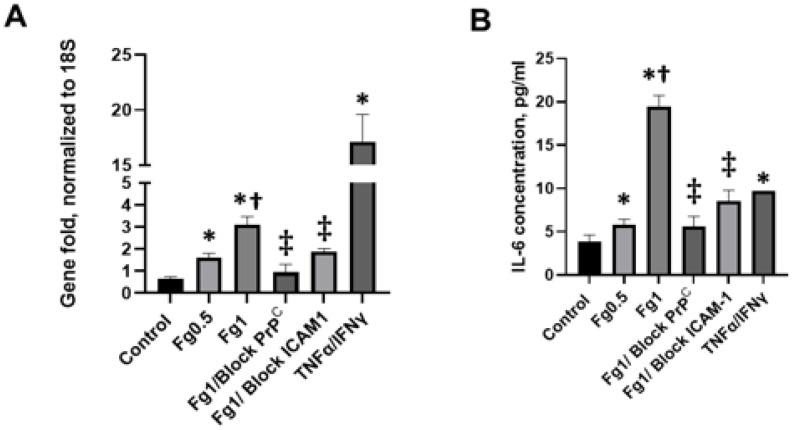
The fibrinogen (Fg)-induced increase in expression of interleukin 6 (IL-6) in neurons. The cells were treated with medium alone (control), 0.5 mg/mL, 1 mg/mL of Fg, 1 mg/mL of Fg in the presence of a cellular prion protein (PrP^C^)-blocking peptide (Fg1/block PrP^C^), or 1 mg/mL of Fg in the presence of a function-blocking antibody against intercellular adhesion molecule-1 (ICAM-1) (Fg1/block ICAM-1) for 24 h. The experimental group treated with tumor necrosis factor alpha (TNFα) co-stimulated with interferon gamma (IFNγ) was used as a positive control. The treated cells were kept for 24 h in 5% CO_2_ at 37 °C. Hirudin was present in all experimental groups to prevent the conversion of Fg to fibrin. (**A**) IL-6 mRNA expression analyzed by quantitative real-time PCR (qRT-PCR). The relative expression of cytokine RNA was normalized to the 18S housekeeping gene. (**B**) The level of IL-6 protein was measured in the medium from neurons using an enzyme-linked immunosorbent assay (ELISA). *p* < 0.05 in all; ∗—vs. Control, †—vs. Fg0.5, ‡—vs. Fg1; n = 3.

Fg dose-dependently upregulated the gene expression of IL-6 (Figure 3A). To further characterize this effect, we tested the level of IL-6 protein present in the media taken from the Fg-treated neurons using an ELISA. Analysis at the mRNA level directly correlated with the results obtained from the measurements of IL-6 protein levels (Figure 3B). Fg-induced an increase in the expression of the IL-6 gene. IL-6 protein expression was reduced with the use of blocking peptide against PrP^C^ or function-blocking antibody against ICAM-1 (Figure 3A,B, respectively).

### 3.4. Fg-Induced Oxidative Stress in Neurons

**Figure 4:**
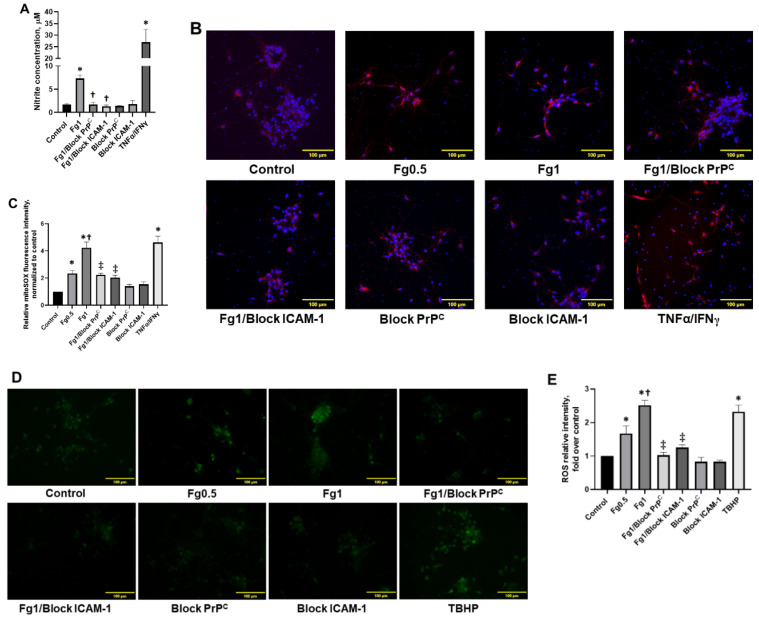
Fibrinogen (Fg)-induced production of nitrite, mitochondrial superoxide, and the generation of reactive oxygen species (ROS) in neurons. The cells were treated for 2 h with medium alone (control), 0.5 mg/mL of Fg, 1 mg/mL of Fg, 1 mg/mL of Fg in the presence of a cellular prion protein (PrP^C^)-blocking peptide (Fg1/block PrP^C^), 1 mg/mL of Fg in the presence of a function-blocking antibody against intercellular adhesion molecule-1 (ICAM-1) (Fg1/block ICAM-1), the prion protein-blocking peptide alone, or the ICAM function-blocking antibody alone. Hirudin was present in all experimental groups to prevent the conversion of Fg to fibrin. (**A**) The graph depicts the concentration of nitrite measured in media collected from treated neurons using a Griess assay. The experimental group with tumor necrosis factor alpha (TNFα) co-stimulated with interferon gamma (IFNγ) was used as a positive control (TNFα/IFNγ). (**B**) Representative images show superoxide production by neuronal mitochondria detected via mitoSOX (red). In these experiments, TNFα/IFNγ was used as a positive control as well. Cellular nuclei were labeled with 4′,6-diamidino-2-phenylindole (blue). (**C**) The summary of the image analyses for the detection of Fg-induced mitochondrial superoxide generation in neurons. (**D**) Representative images show ROS generation by neurons. *Tert*-butyl hydroperoxide (TBHP), a common inducer of ROS production, was used as a positive control in this assay. (**E**) The summary of image analyses for the detection of Fg-induced ROS generation in neurons. *p* < 0.05 in all; ∗—vs. Control, †—vs. Fg0.5 and ‡—vs. Fg1; n = 4.

Fg increased nitrite production in neurons (Figure 4A). In addition, Fg dose-dependently increased the production of mitochondrial superoxide in neurons, which was defined through the quantification of the fluorescence intensity of MitoSOX red (Figure 4B,C) and the generation of intracellular reactive oxygen species (ROS) (Figure 4D,E). All nitrate levels, mitochondrial superoxide, and intracellular ROS were reduced in neurons with blocking peptide against PrP^C^ or function-blocking antibody against ICAM-1 (Figure 4A–C, and D,E, respectively). Production of neuronal nitrite and mitochondrial superoxide with TNFα/IFNγ and intracellular ROS with tert-butyl hydroperoxide (TBHP) confirmed positive responses of neurons to the activators (Figure 4A–C, and D,E, respectively).

## 4. Discussion

Proinflammatory effects of excess Fg, such as increase in vasoconstriction, vascular permeability, and neuron, astrocyte and other glial cell activation, are well documented [3,25,28,29]. We have shown the functional effects of extravasated Fg in brain, such as neurodegeneration and reduction in STM during m-mTBI. However, beyond the functional implications of Fg-activated astrocytes on alterations of neurons [4,24], the direct interaction of Fg or fibrin with neurons and the mechanisms leading to neuronal alterations are not clear.

We have previously demonstrated that Fg could extravasate from non-ruptured cerebral microvessels and deposit in extravascular space during m-mTBI [4]. These effects were associated with reduction in STM [3,4], a detriment, which in part, could have been a direct effect of the extravasated soluble Fg on neurons as we found in the present study. To confirm this notion, first we tested if soluble Fg had deleterious effects on neurons or if this is a characteristic specific to fibrin-neuron interactions.

A neuron live/dead test was performed on Fg-treated neurons in the presence or absence of hirudin, an anti-thrombin anticoagulant (thrombin inhibitor). Data showed no significant difference in the rate of cell death induced by Fg in the presence or absence of hirudin, suggesting that soluble Fg acts similarly to its derivative, fibrin, in the time period of the experiment. These data indicate that before its conversion to fibrin by thrombin, Fg could have a significant effect on neurons. Therefore, as we established that soluble Fg can affect neurons, all the following experiments were performed in the presence of hirudin. This allowed us to define specific effects of soluble Fg, which, in vivo, was found to be extravasated from brain microvessels due to inflammation caused by TBI as we have seen in our previous studies [3,4,5]. Studies by others that focus on effects of soluble Fg on neuronal tissue are rare. Our data indicate that the neuronal death that is caused by soluble Fg is specific, and it occurs as a result of an interaction between Fg and its receptors, PrP^C^ and ICAM-1, on the surface of neurons. This is the first time that a functional effect resulting from the interaction between soluble Fg and neurons has been reported. Similar to what we found, Fg has been shown to increase cell death in the SH-SY5Y neuroblastoma cells, a cell type that displays a similar phenotype as neurons [30]. Fibrin deposition has been associated with neuronal loss and a reduced density of neurons in postmortem human brain samples with multiple sclerosis (MS) [31] and TBI [32]. Combined, these results indicate that Fg and, at later stages, fibrin can affect neurons and alter their function if they have direct contact with each other.

Fibrin deposits have been found postmortem in the brains of patients with TBI [2,32,33], Alzheimer’s disease (AD) [34], and MS [31]. Furthermore, plaque formations containing Fg/fibrin were found in brains with inflammatory neurodegenerative diseases associated with memory reduction such as AD [35], MS [36], and TBI [37]. The proinflammatory role of Fg/fibrin is manifested in its ability to bind to certain integrin receptors such as CD11b/CD18 and CD11c/CD18 through a ligand–receptor interaction in order to activate a wide range of immune cells [29]. However, direct interactions between Fg and PrP^C^ or ICAM-1 on neurons have never been demonstrated. We found that, during TBI, extravasated Fg was deposited in the vasculo–astrocyte interface and visibly co-localized with astrocytes [4]. We showed previously that Fg was strongly associated with astrocytic PrP^C^ and ICAM-1, suggesting that it was binding to these receptors on the surface of astrocytes [24]. Since there is a very low probability that Fg would reach neurons at the same concentration that exists in the blood (~2 mg/mL, or ~4 mg/mL during inflammation) after extravasation, we used Fg at a concentration of 1 mg/mL or lower in our present study to test the direct effects of Fg on neurons. In the present study, we showed that Fg was positively associated with neuronal PrP^C^ and ICAM-1. This is the first time that we documented a specific interaction between Fg and its receptors on neurons which were visualized with the highly specific and sensitive method, PLA. The data suggest that Fg can directly interact with neurons via its receptors, which are distributed all along the cell. However, based on our observations, there were greater associations of 1 mg/mL of Fg to astrocytic PrP^C^ (1153 ± 221 PLA signal/cell) (Mean ± SD) and ICAM-1 (586 ± 193 PLA signal/cell) seen in our previous study [24] when compared to the association of Fg with neuronal PrP^C^ (142 ± 27 PLA signal/cell) and ICAM-1 (146 ± 10 PLA signal/cell). We speculate that this may be the result of a possible differences in the number of the Fg receptors on astrocytes vs. those on neurons. The data show that blocking PrP^C^ or the function of ICAM-1 with a blocking peptide/antibody (respectively) reduced the intensity of Fg’s interaction with neurons, suggesting a specificity of Fg–neuron interaction.

We have previously demonstrated that Fg induced an increase in the expression of IL-6, along with other proinflammatory cytokines such as the C-X-C motif chemokine ligand 10 and chemokine ligand 2, in astrocytes [24]. These data suggest that Fg induces the activation of astrocytes with functional polarization towards their neurotoxic A1 phenotype [38,39]. The activation of astrocytes causes the release of inflammatory cytokines and the generation of ROS that most likely exacerbates inflammatory conditions typically found during TBI [24,40]. We found that the direct effect of Fg on neurons also causes an increase in IL-6 expression in these cells. It has been well documented that IL-6 was increased in cases of TBI found in human patient samples [41,42] and animal models of TBI [43]. The maximum IL-6 concentrations in the serum of TBI patients were correlated with peak levels of acute-phase proteins, including C-reactive protein and Fg [42]. Interestingly, in the same study, it was found that a higher IL-6 concentration was detected in the cerebrospinal fluid (CSF) when compared to that in serum, suggesting that IL-6 may be produced predominantly in the central nervous system [42]. The increased IL-6 found in the CSF may be the result of the activation of several cell types including Fg-activated astrocytes, as we have found previously [5], and neurons, as shown in the present study. We defined the activation of neurons as an ability of these cells to overexpress the proinflammatory cytokine IL-6 and produce oxidative stress responses.

ROS, which is necessary for the normal cell function, is continuously generated during the process of respiration [44]. However, when an excess of ROS is generated or the antioxidant system is overwhelmed, it could result in the alteration of membrane lipids, proteins, and nucleic acids, creating oxidative stress that leads to the detrimental effects of ROS [44,45], which are associated with neurodegenerative disorders [46].

The involvement of fibrin [47] and Fg [48,49] in the generation of ROS in microglia and the causative factor of oxidative damage in the brain has been well documented [47,48,49]. We recently reported that Fg induced an increased generation of ROS in astrocytes [24]. In the present study, we showed for the first time that Fg dose-dependently increased the generation of ROS, mitochondrial superoxide, and nitrite in neurons. Both the increase in IL-6 and the increased oxidative damage were ameliorated when treated with a blocking peptide for PrP^C^ or the antibody against ICAM-1, indicating that Fg binding to PrP^C^ and/or ICAM-1 is in part responsible for the proinflammatory responses and the possible subsequent signaling. Others have shown that fibrin’s interaction with CD11b/CD18 stimulates the proinflammatory cytokine interleukin-1 beta in peripheral blood mononuclear cells [50]. This is in agreement with our previous [24] and current findings indicating that Fg’s interactions with its receptors, such as PrP^C^ and ICAM-1, resulted in the expression of proinflammatory cytokines and oxidative damage.

Others have shown that PrP^C^ is involved in cell-redox homeostasis through ROS production via dinucleotide phosphate oxidase (NAPDH) and extracellular regulated kinases 1/2 (ERK-1/2) signaling stress [51]. In that study, antibody-mediated ligation was used to mimic an extracellular signal acting on PrP^C^, which resulted in NADPH oxidase-dependent ROS production in neuronal, hypothalamic, and lymphoid cells [51]. This PrP^C^ ligation-induced ROS formation results in neuronal and other brain cell toxicity, as well as oxidative stress [51]. Therefore, the Fg and PrP^C^ interaction shown here could be a source of ROS formation possibly via NADPH and/or ERK-1/2 signaling [24,51].

It has been shown that an intravenous administration of Fg results in the activation of microglia, neuronal dendritic loss, and dendritic spine elimination, all being associated with cognitive decline [49]. Inhibition of Fg synthesis reduced the expression of activating transcription factor 3, a marker of inflammation, and decreased the expression of PrP^C^, Fg deposition, and the formation of Fg–PrP^C^ complexes in the brain [5]. These effects resulted in improvement of vascular cognitive impairment during TBI in mice [5]. Fg–amyloid beta and Fg–PrP^C^ complexes are associated with reduced cognitive function and STM in patients and mice, respectively [3,37]. Inflammatory conditions that develop during TBI that are associated with extravasation of Fg, which will eventually be converted to fibrin, possibly contribute to plaque formation and lead to the subsequent neurodegeneration that may be the result of a direct interaction of Fg with its neuronal receptors PrP^C^ and ICAM-1, an increased expression of the inflammatory cytokine IL-6, and the generation of ROS.

In summary, our study shows that not only does fibrin cause neuronal death, as it has been widely accepted, but soluble Fg can as well. This effect may be the result of an interaction between Fg and neurons via its neuronal receptors PrP^C^ and ICAM-1. Our findings further imply that Fg’s interaction with neurons is, in part, responsible for Fg-induced expression of proinflammatory cytokine IL-6 in neurons and the oxidative damage that ultimately results in neuronal death, which most likely results in the neurodegeneration during m-mTBI seen in our previous study [4].

## Supplementary Materials

The following are available online at https://www.mdpi.com/article/10.3390/biom11091381/s1, Figure S1: Neuronal cell death induced by fibrinogen (Fg) and fibrin, Figure S2: Interactions of fibrinogen (Fg) with its receptors, cellular prion protein (PrP^C^) and intercellular adhesion molecule-1 (ICAM-1), on the surfaces of neurons detected by proximity ligation assay (PLA), Figure S3: Homogeneity of neuronal cell culture.

## Abbreviations

AD	Alzheimer’s disease
TBI	traumatic brain injury
PrP^C^	cellular prion protein
STM	short-term memory
ICAM-1	intercellular adhesion molecule-1
IL-6	interleukin 6
ROS	reactive oxygen species
Fg	fibrinogen
m-mTBI	mild-to-moderate traumatic brain injury
TNFα	Tumor Necrosis Factor alpha
IFNγ	interferon gamma
PLA	proximity ligation assay
IgG	Immunoglobulin G
ELISA	enzyme-linked immunosorbent assay
qRT-PCR	quantitative real-time PCR
TBHP	*tert*-butyl hydroperoxide
MS	multiple sclerosis
CSF	cerebrospinal fluid
NAPDH	dinucleotide phosphate oxidase
EthD-1	ethidium homodimer-1
AOI	area of interests
ANOVA	analysis of variance
