# Supplementary material for: The Effects of Fibrinogen’s Interactions with Its Neuronal Receptors, Intercellular Adhesion Molecule-1 and Cellular Prion Protein
Source: Biomolecules. 2021 Sep 18;11(9):1381. doi: 10.3390/biom11091381 (PMC8464854; doi:10.3390/biom11091381)
Supplement: Supplementary file 1 [file biomolecules-11-01381-s001.zip › biomolecules-1348206-supplementary.pdf]

**Supplementary Materials**

## 1. Results and Discussion

### *1.1. Neuronal death by soluble fibrinogen (Fg) or by fibrin*

We found that Fg dose-dependently increased neuronal death regardless of the presence or absence of hirudin that was used to inhibit thrombin and thus prevent the conversion of soluble Fg to fibrin (Figure S1). Our data indicated that soluble Fg had toxic effects on neurons, and neuronal cell death did not differ whether it was induced by soluble Fg or fibrin (Figure S1). Based on these results, to specifically define the effects of soluble Fg, all further experiments were performed in the presence of hirudin.

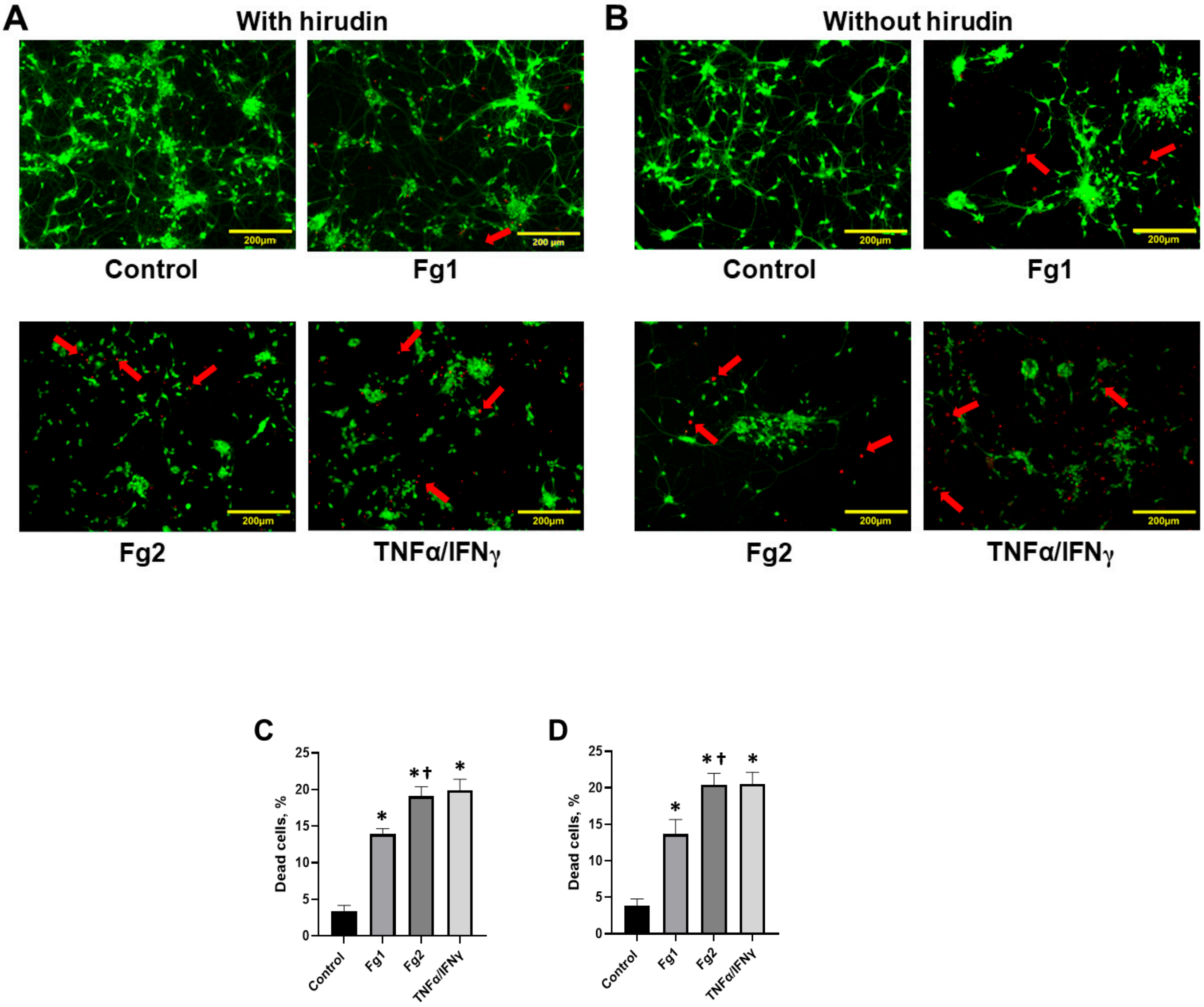


**Figure S1. Neuronal cell death induced by fibrinogen (Fg) and fibrin.** Representative images show staining of live/dead neurons that were treated with medium alone (control), 1 mg/ml of Fg (Fg1), or 2 mg/ml of Fg (Fg2) in the presence (**A**) or absence (**B**) of hirudin for 24 hours. The treatment with Tumor Necrosis Factor alpha (TNFα)/interferon gamma (IFNγ) was used as a positive control. (**C and D**) Summary of image analyses for the detection of dead neurons in the presence or absence of hirudin, respectively. An automatic cell count was performed based on the fluorescence signal threshold of images provided by the CellSens Dimension software. Data are presented as number of dead cells as a percent of a total number of cells in the selected constant area of interest and averaged for each experimental group. $P<0.05$; ∗ - vs. Control, † - vs. Fg1; n=4.

## *1.2. Interaction of Fg with neuronal cellular prion protein ($PrP^C$) and intercellular adhesion molecule-1 (ICAM-1)*

Here we present the results of some control experiments performed to validate the specificity of our proximity ligation assay (PLA) that was used to determine association of Fg with its neuronal receptors $PrP^C$ and ICAM-1. Thus, in addition to using the pairing of IgG with the Fg antibody as an isotype control (Figure 2E), PLAs were also performed with the omission of the $PrP^C$ blocking peptide, the antibody against ICAM-1, or the antibody against Fg to detect non-specific binding of each of the primary antibodies. There were no PLA signals detected in any of the three experimental groups mentioned above (Figure S2). These results suggest a high specificity of the used antibodies in our experiments and validate of the PLA results shown in Figure 2.

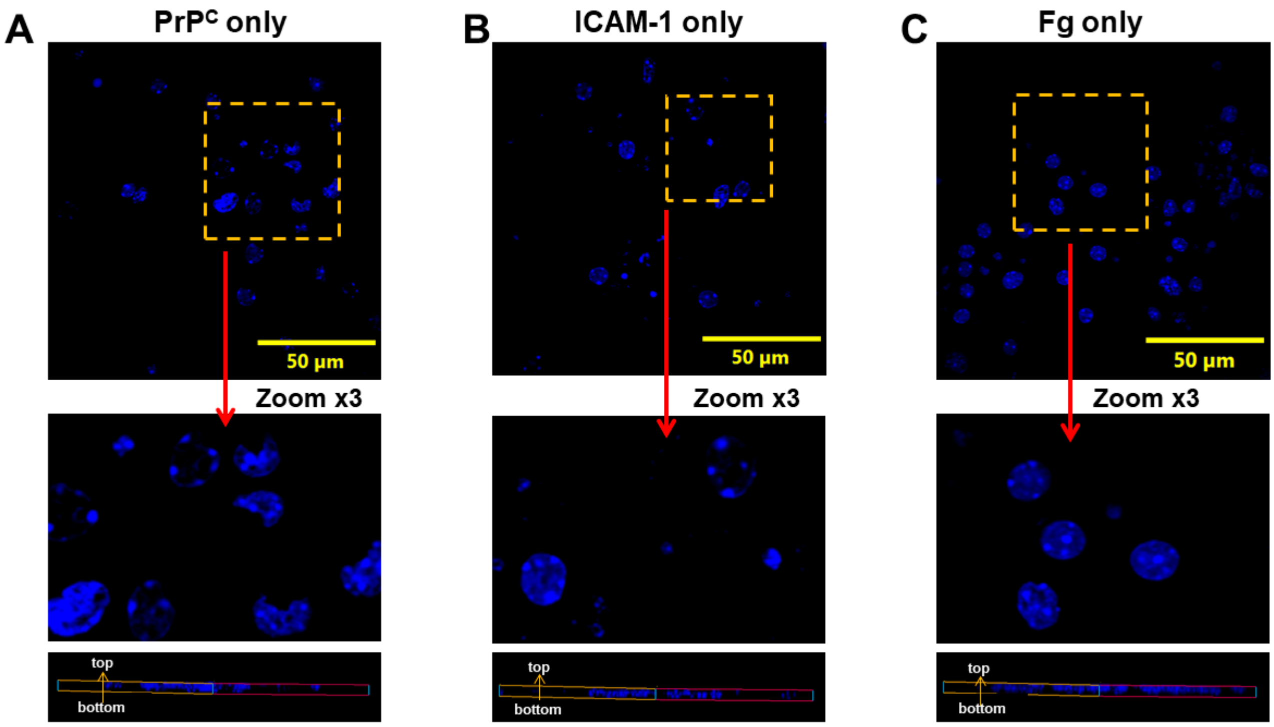


**Figure S2. Interaction of fibrinogen (Fg) with its receptors, cellular prion protein (PrP$^C$) and intercellular adhesion molecule-1 (ICAM-1), on the surface of neurons detected by proximity ligation assay (PLA).** Representative images that lack PLA signals (if PLA signal were present, they would have been shown in red) depicting interactions between Fg and neuronal ICAM-1 or PrP$^C$. Neurons were treated with 1 mg/ml of Fg for 24 hours. As control groups, interactions of Fg and its receptors were detected using only one of the two required antibodies: anti-PrP$^C$ (**A**), anti-ICAM-1 (**B**), or anti-Fg (**C**). Cellular nuclei were labeled with 4′,6-diamidino-2-phenylindole in blue.

## *1.2. Homogeneity of neuronal cell culture*

Here we present the results of immunofluorescence staining of cultured neuronal cells maintained in medium for 7, 10 and 13 after seeding. The cells were fixed with 4% paraformaldehyde in phosphate buffer solution (PBS) for 15 minutes, rinsed with PBS before permeabilization with 0.1% Triton X-100 in PBS for 15 minutes. Five % goat serum in PBS was used for blocking step for 2 hours at room temperature. Primary antibodies used were against

microtubule associated protein 2 (Invitrogen, cat. # PA1-10005) at 1:500 dilution (host species, chicken) and glial fibrillary acidic Protein (ProteinTech, Chicago, IL cat. # 16825-1-AP) at 1:200 dilution (host species, rabbit). Cells were incubated with primary antibodies in 1% goat serum in PBS overnight at 4°C. Secondary antibodies used were Alexa Fluor goat-anti chicken 488 and goat anti-rabbit 594, both at 1:200 dilution in 1% goat serum in PBS. Cells were incubated with these antibodies for 2 hours at room temperature. ProLong™ Diamond antifade mountant with 4′,6-diamidino-2-phenylindole was used as mounting media (Invitrogen, cat. # P36962). Images showed that there were minimal glial cells detected compared to population of neurons assessed at day 7 (A), day 10 (B), and day 13 (C) of observation (Figure S3).

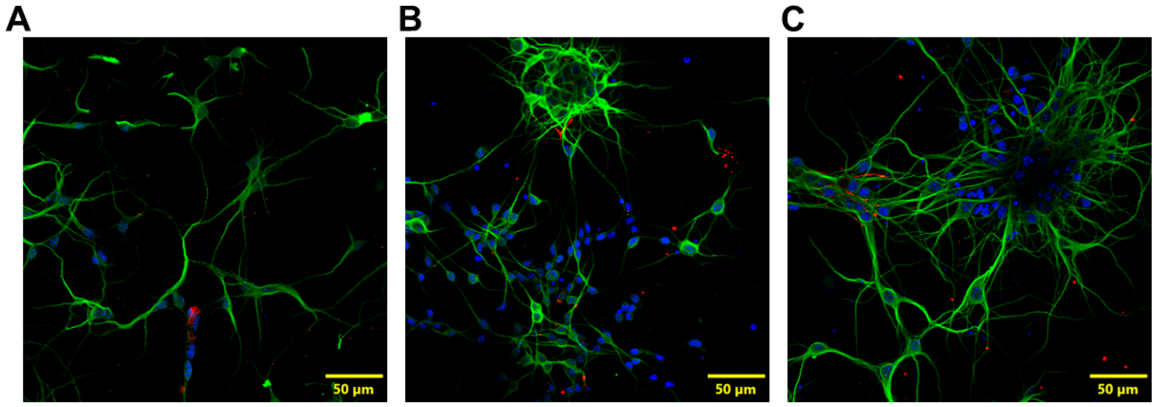


**Figure S3.** Homogeneity of n**euronal cell culture.** Representative images of cultured neurons stained with antibodies against microtubule associated protein 2 (Green) and glial fibrillary acidic protein (Red). Neurons were fixed and stained on day 7 (**A**), 10 (**B**) and 13 (**C**) after seeding. Cellular nuclei were labeled with 4′,6-diamidino-2-phenylindole in blue.
